# Regression Models for Log-Normal Data: Comparing Different Methods for Quantifying the Association between Abdominal Adiposity and Biomarkers of Inflammation and Insulin Resistance 

**DOI:** 10.3390/ijerph110403521

**Published:** 2014-03-27

**Authors:** Sara Gustavsson, Björn Fagerberg, Gerd Sallsten, Eva M. Andersson

**Affiliations:** 1 Occupational and Environmental Medicine, Sahlgrenska University Hospital and Academy, University Of Gothenburg, Gothenburg SE-405 30, Sweden; E-Mails: gerd.sallsten@amm.gu.se (G.S.); eva.m.andersson@amm.gu.se (E.M.A.); 2 Wallenberg Laboratory, Sahlgrenska Center for Cardiovascular and Metabolic Research, Sahlgrenska University Hospital, Gothenburg SE-413 45, Sweden; E-Mail: bjoern.fagerberg@wlab.gu.se; 3 Department of Molecular and Clinical Medicine, University of Gothenburg, Gothenburg SE-413 45, Sweden

**Keywords:** linear regression model, log-normal distribution, heteroscedasticity, biomarkers of inflammation, insulin resistance, simulation study

## Abstract

We compared six methods for regression on log-normal heteroscedastic data with respect to the estimated associations with explanatory factors (bias and standard error) and the estimated expected outcome (bias and confidence interval). Method comparisons were based on results from a simulation study, and also the estimation of the association between abdominal adiposity and two biomarkers; C-Reactive Protein (CRP) (inflammation marker,) and Insulin Resistance (HOMA-IR) (marker of insulin resistance). Five of the methods provide unbiased estimates of the associations and the expected outcome; two of them provide confidence intervals with correct coverage.

## 1. Introduction

A common objective in medical research is to identify and quantify associations. For example, this could include evaluating a biomarker or estimating personal exposure levels based on questionnaires and occupational history. In these cases regression analysis is often used. It can also be important to estimate the expected value, e.g., the expected exposure. A person’s risk of developing an exposure-caused disease is related to the dose, and the dose is usually estimated by the cumulative exposure. In group-based exposure assessment, the arithmetic mean is considered superior to the geometric mean, as a dose-related variable [1,2]. The arithmetic mean is also preferred, in the form of mean exposure for individuals over time, when assessing long-term effects of exposures [3].

Many biological variables (e.g., exposure and biomarkers) have a skewed distribution with a median smaller than the mean and only positive values. It is also common with heteroscedasticity, where the variance increases with the expected value. Such data can often be described by a log-normal or quasi-log-normal distribution [4,5,6]. A common way to analyze a log-normal variable *Y* is to log-transform (*Z* = ln(*Y*)) so that Z follows a normal distribution with expected value *μ_z_* and standard deviation *σ_z_*. The geometric mean of *Y* is then found as exp(*μ_z_*), while the expected value of *Y* (the arithmetic mean) is found as *μ*_Y_ = 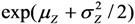. In cases where the expected value *μ*_Y_ depends on several predictors, regression analysis is often based on the log-transformed data, *Z* = 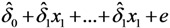, and the expected value of *Y* is estimated as 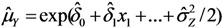. This produces effect-measures on the multiplicative scale and the interpretation is that *Y* is expected to increase 100(exp(*δ_i_*) − 1) percent as *x_i_* increases one unit, see e.g. [7]. 

We investigated the situation where we want an estimate of the absolute effect, thus we need the model to be linear on the original scale, 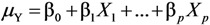, in order to produces effect-measures on the additive scale. This is of interest e.g., in exposure modeling, when exposure time is an important factor and it is reasonable that the effect of time on exposure is linear. Effect-measures on the additive scale have also been discussed in relation to statistical *vs*.biologic interaction. Biologic interaction occurs when the effect of one cause depends on the presence of another cause, e.g., environmental causes and genetic predisposition, and is often defined as departure from additivity [8,9].

Different regression methods, suitable for log-normal data, were investigated and the aim was to estimate the absolute effect β*_i_* of each predictor. Because of the heteroscedasticity, the ordinary least squares regression will produce erroneous tests and confidence intervals. One solution is to use a weighted least squares regression. Another way to handle non-normal distributions is to use a general linear model, GLM, in which the distribution of the response variable *Y* belongs to the natural exponential family and the expected value of *Y* is linked to a linear model by a link function, g(*μ*_Y_) = β_0_+ β_1_*X*_1_ + ...+ β_p_*X*_p_, see [10]. One example of a GLM that is suitable for the log-normal distribution is the gamma distribution with an identity link. Another possibility is the normal distribution and an exponential link, applied to *Z* = ln(*Y*).

We compared the different regression methods using both large scale simulations and by applying them to a cross-sectional data set with the aim to quantify the association of abdominal adiposity with inflammation and insulin resistance (two well-known associations).

## 2. Linear Regression with a Lognormal Response

We considered a regression model where the expected value of a continuous log-normal response variable *Y* is a linear function of the predictors *X*_1_,*X*_2_,..*X*_p_ :

*μ*_Y_ = β_0_ + β_1_*X*_1_ + … + β*_p_X_p_* (1)

The variance of *Y* depends on both the expected value of *Y*, *μ_Y_*, and the variance of *Z* = ln(Y), 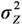; 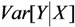 = 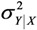 = 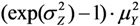. 

Ordinary least squares regression (here denoted LS_lin_) can be used to obtain unbiased estimates 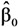, 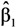, …, 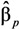 However, the estimates provided by LS_lin_ assume homoscedasticity, which, as previously noted, is incorrect for a log-normal variable. This incorrect variance assumption leads to incorrect statistical inferences. 

In a situation with heteroscedasticity, weighted least squares regression (here denoted WLS) can be used. WLS can account for the heteroscedasticity by weighting each observation, *Y*_i_, with the inverse of its variance, 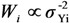. For a log-normal distribution, the weight for *Y*_i_ is 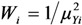, where LS_lin_ can provide estimates of *μ_Yi_*. Unlike LS_lin_, WLS provides an estimate of the variance 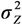. 

When the response *Y* is log-normally distributed, data are often log-transformed, ln(*Y*) = *Z*, and a log-linear model is estimated:

*μ_Z_*_ǀ*X*_ = *δ*_0_ + *δ*_1_*X*_1_ + … + *δ_p_X_p_* (2)

where the expected value of *Y* is 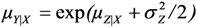. Ordinary least squares regression on *Z* (here denoted LS_exp_) provides estimates of the relative effect (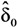, 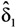, …, 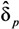) as well as an estimate of the variance 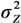 but no estimates of the absolute effects. Thus, both (1) and (2) can be used to estimate *μ*_Yǀ*X*_ and *σ_Z_*. The reason for including LS_exp_, even if the linear model in (1) is assumed, is that LS_exp_ is commonly used for log-normal data.

The log-normal distribution is often approximated by the gamma distribution, with parameters *μ* (expected value) and *ν* (scale parameter, Var[*Y*] = *μ*^2^/*ν*). A generalized linear model (GLM) with gamma distribution and the identity link (denoted GLM_G_), provides estimates 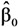, 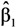, …, 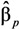 and an estimate of 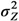 can be found through the transformation 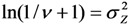. 

Another GLM that can be used to estimate the absolute effects is one with a normal distribution and the link function exp(*), applied to *Z* = ln(*Y*), here denoted GLM_N_, such that

exp(*μ_Z_*_ǀ*X*_ ) = *ϕ*_0_ + *ϕ*_1_*X*_1_ + … + *ϕ_p_X_p_* (3)

The expected value of *Y* is then found as 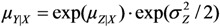.

The method GLM_N_, does not, however, take into account the stochastic variation due to estimating 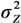. Therefore we also used a maximum likelihood method (ML_LN_, see [11,12]) based on the likelihood function of the log-normal distribution:

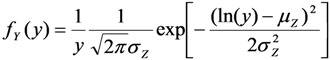
(4)
where 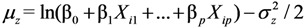. The estimates 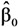, 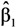, …, 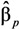 and 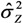 are found using iterations, for example the Newton-Raphson iteration used here [13]. 

### 2.1. Confidence Intervals

For LS_lin_, WLS, GLM_G_ and ML_LN_, a 95% confidence interval for *μ*_Yǀ*X*_ is estimated as 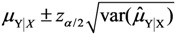, where the sample-specific variance is estimated as:

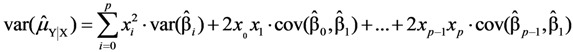
(5)
where *x*_0_ = 1, 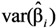 and 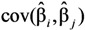 are the sample-specific estimates of the variance and the covariance (the sample-specific standard error is 
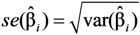
). 

For GLM_N_, a confidence interval is estimated as 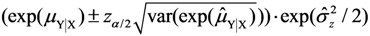, where the sample-specific variance of the linear estimator is estimated as:

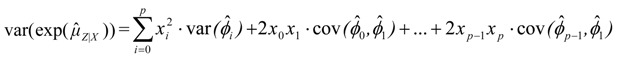
(6)

For LS_exp_, a confidence interval for *μ_Y_*_ǀ*X*_ is estimated as 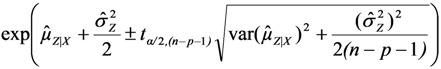, using the modified Cox method [14]. The sample-specific variance is estimated as:

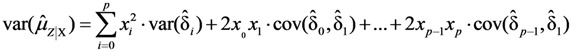
(7)
where *x*_0_ = 1, 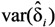 and 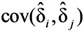 are the sample-specific estimates of the variance and the covariance. 

### 2.2. Simulation Model

In a simulation study we compared the large-sample properties of the methods for estimating the expected value of *Y* and the effect of each predictor, when data follow a log-normal distribution. To obtain a realistic scenario, a simulation model was estimated from a real-life data set on personal exposure to PM_2.5_-particles in Sweden. These data are described in [15]. PM_2.5_ is the mass (microgram/m^3^) of particles smaller than 2.5 micrometers, which implies that they are small enough to bypass the respiratory defenses and enter into the lungs. Increased levels of PM_2.5_ have been associated with increased mortality from cardiovascular disease and lung cancer [16,17]. Several sources contribute to the personal exposure to PM_2.5_, two of them are tobacco smoke and traffic exhaust [18].

The expected outcome, personal exposure to PM_2.5_-particles (*μ*g/m^3^), was assumed to be a linear function of the number of cigarettes per day, Smoke, and residential outdoor concentration of PM_2.5_ (*μ*g/m^3^), ConcOut:

E[Y] = *μ*_Y_ = 1.564 +0.122·*Smoke* + 0.075·*ConcOut* (8)

Observations were then simulated according to the model *Z* = ln(*μ_Y_*)-0.383^2^/2 + *ε*, where ε~N(0, σ_Z_ = 0.383). In order to facilitate interpretation and comparison without the introduction of unnecessary variation, balanced data were used in the simulations, with the following values of the explanatory variables: ConcOut = {2, 8, 14}, Smoke = {0, 7, 14}. Thus we estimated the expected PM_2.5_ exposure for 9 combinations of outdoor concentration and cigarettes smoked. Simulations with 10,000 replicates were used to evaluate the potential bias in the estimates of β_0_, β_1_ and β_2_, the sample-specific standard error 
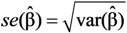
 as well as the true standard deviation 
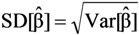
 and also the properties of confidence intervals for *μ*_Y_. 

### 2.3. The DIWA Data Set

The DIWA dataset is a population-based cohort of 64-year-old women from the city Gothenburg in Sweden and has previously been described in detail in [19]. Of the 2,595 women who was screened 9.5% had diabetes mellitus (DM) [20], and of these 230 participated in the study, together with similar sized, randomly-selected groups of women with impaired glucose tolerance (IGT, *n* = 209) and normal glucose tolerance (NGT, *n* = 190). The World Health Organization criteria for capillary glucose cut-off values were used to define diabetes and impaired glucose tolerance [21]. Insulin resistance was also assessed, as well as a large number of biomarkers including high sensitivity C-reactive protein (hS-CRP). The examination also included a questionnaire regarding medical history and lifestyle factors, including smoking habits (never smoker, past smoker and smoker) and recreational physical activity (<2 h/week and ≥2 h/week). Body weight and waist circumference were also measured. 

CRP is an acute-phase protein found in blood serum and its levels increase during an inflammatory process. CRP is mainly used as an inflammatory marker in clinical practice and should, for a healthy person, be less than 5 mg/L. Diabetes, smoking, obesity and insulin resistance are all been associated with small increases in CRP-levels as assessed by high sensitivity methods [22,23,24,25]. 

Insulin resistance is a condition where the body has a reduced ability to respond to the insulin hormone which can cause blood glucose to rise above normal levels. Insulin resistance can lead to type 2 diabetes and cardiovascular disease. Even if insulin resistance is most common among persons with diabetes mellitus of type 2 or impaired glucose tolerance, it is also present in about 25% of non-obese persons with normal glucose tolerance, [26]. Obesity, and in particular abdominal obesity, is associated with increased insulin resistance [27,28]. Other factors are smoking and low physical activity [29,30]. In our study, insulin resistance was measured using the homeostasis model assessment of insulin resistance (HOMA-IR), which is a mathematical formula for quantifying insulin resistance [31]; HOMA-IR is the product of fasting serum glucose and fasting serum insulin (fasting serum glucose (mmol/L)∙fasting serum insulin/22.5). A cut-off value around 2.5 is often used as an upper limit for normal HOMA-IR [32,33,34,35].

## 3. Results

### 3.1. Bias and Standard Deviation of the Regression Coefficients (Simulation Study)

In the simulation study, balanced data sets were computer-generated using the model in Section 2.2, with two explanatory variables (Smoke and ConcOut) each with three levels. To obtain a balanced sample with at least 100 observations, the sample size *n* = 108 was used. For each sample, coefficients of the regression model were estimated, along with the expected outcome (personal exposure) and its confidence interval. 

**Table 1:** Estimates of the regression coefficients; expected value of the estimate, E[*], true standard deviation of the estimated coefficient, SD[*], and expected sample specific standard error, E[*se*(*)]. The true coefficient values are β_0_ = 1.564, β_1_ = 0.122, β_2_ = 0.075, σ_Z_ = 0.383. Results of the simulation study for sample size *n* = 108 (*r* = 10,000 replicates).

		LS_lin_	WLS	ML_LN_	GLM_G_	GLM_N_^1^	LS_exp_^2^
Intercept							
	E[*]	1.566	1.560	1.563	1.565	1.567	0.487
	SD[*]	0.226	0.190	0.183	0.187	0.180	0.083
	E[*se*(*)]	0.269	0.187	0.180	0.178	0.179	0.084
Parameter for X_1_							
	E[*]	0.121	0.122	0.122	0.122	0.121	0.042
	SD[*]	0.021	0.019	0.019	0.020	0.019	0.006
	E[*se*(*)]	0.021	0.019	0.018	0.018	0.018	0.006
Parameter for X_2_							
	E[*]	0.075	0.075	0.075	0.075	0.075	0.027
	SD[*]	0.024	0.021	0.021	0.021	0.02	0.008
	E[*se*(*)]	0.024	0.021	0.020	0.020	0.02	0.008
E[ 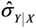 ]		1.229					
SD[ 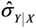 ]		0.143					
Scale parameter					7.330	0.377	
SD[scale parameter]					1.015	0.026	
E[ 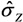 ]			0.379	0.376	0.358 ^3^	0.377	0.384
SD[ 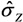 ]			0.031	0.026	-	0.026	0.026

^1^ After transformation of the coefficients in eq (3): 
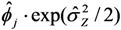
 and 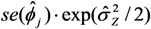; ^2^ Coefficients 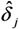 estimated under assumption of a log-linear model; ^3^ After transformation: 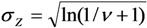.

All methods except LS_exp_ provided unbiased estimates of the regression coefficients. Among the absolute-effects methods, GLM_N_ tended to have the best precision (smallest SD). The sample-specific standard errors, *se*, were close to the true standard deviations, SD. All methods except LS_lin_ provided reasonable estimates of *σ_Z_*, although the transformed scale parameter from GLM_G_ was too small (Table 1).

All methods except LS_exp_ provided an unbiased estimate of the expected value. The interval length was similar between WLS, ML_LN_, GLM_G_ and GLM_N_, but tended to be smaller for the two GLM methods (Table 2).

**Table 2:** Estimated expected value and expected length of 95% confidence interval for 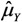, for a sample of *n* = 108 observations (results from simulation with *r* = 10,000 replicates).

Expected value	E[ 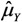 ]						E[length]					
*μ*_Y_	LS_lin_	WLS	ML_LN_	GLM_G_	GLM_N_	LS_exp_	LS_lin_	WLS	ML_LN_	GLM_G_	GLM_N_	LS_exp_
1.714	1.72	1.71	1.71	1.72	1.72	1.85	0.927	0.631	0.609	0.594	0.6	0.544
2.164	2.17	2.16	2.16	2.17	2.17	2.17	0.733	0.533	0.518	0.501	0.507	0.506
2.614	2.62	2.61	2.61	2.62	2.62	2.55	0.927	0.825	0.797	0.774	0.783	0.749
2.568	2.57	2.57	2.57	2.57	2.57	2.49	0.733	0.605	0.588	0.567	0.574	0.58
3.018	3.02	3.02	3.02	3.02	3.02	2.91	0.464	0.467	0.462	0.437	0.443	0.439
3.468	3.47	3.47	3.47	3.47	3.47	3.42	0.733	0.763	0.743	0.715	0.723	0.798
3.422	3.42	3.42	3.42	3.42	3.42	3.34	0.927	0.950	0.920	0.89	0.9	0.982
3.872	3.87	3.87	3.87	3.87	3.87	3.92	0.733	0.850	0.827	0.796	0.804	0.914
4.322	4.32	4.32	4.32	4.32	4.32	4.60	0.927	1.026	0.997	0.962	0.972	1.351

LS_lin_ had the largest standard deviation, especially for small and large values of *μ*_Y_. Among the methods that provided an unbiased estimate of *μ*_Y_, GLM_N_ had the smallest standard deviation. For all methods except LS_lin_, the sample-specific standard error tended to be an underestimation (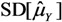
> E[*se*(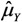)]), Table 3.

**Table 3:** True standard deviation and sample-specific standard error for the 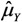-values; SD[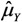] = 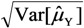 and *se*(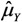) = 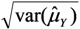. Results from simulation with *n* = 108 observations, *r* = 10,000 replicates.

Expected value	SD[ 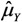 ]						E[*se*( 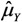 )]					
*μ*_Y_	LS_lin_	WLS	ML_LN_	GLM_G_	GLM_N_	LS_exp_	LS_lin_	WLS	ML_LN_	GLM_G_	GLM_N_	LS_exp_
1.714	0.191	0.161	0.156	0.159	0.154	0.136	0.238	0.159	0.154	0.152	0.154	-
2.164	0.145	0.135	0.132	0.135	0.132	0.128	0.188	0.135	0.131	0.128	0.13	-
2.614	0.220	0.209	0.202	0.211	0.205	0.190	0.238	0.208	0.201	0.198	0.201	-
2.568	0.167	0.153	0.150	0.154	0.151	0.147	0.188	0.153	0.148	0.145	0.147	-
3.018	0.121	0.118	0.118	0.120	0.120	0.112	0.119	0.118	0.117	0.112	0.113	-
3.468	0.210	0.195	0.190	0.196	0.192	0.204	0.188	0.192	0.187	0.183	0.185	-
3.422	0.251	0.241	0.234	0.244	0.238	0.251	0.238	0.240	0.232	0.228	0.231	-
3.872	0.228	0.217	0.212	0.219	0.215	0.235	0.188	0.214	0.209	0.204	0.206	-
4.322	0.290	0.263	0.256	0.264	0.258	0.345	0.238	0.259	0.251	0.246	0.249	-

All methods except LS_lin_ and LS_exp_ provided coverage close to the nominal, but both GLM_G_ and GLM_N_ tended to give too low coverage, whereas ML_LN_ was slightly better. Using LS_lin_ resulted in too high coverage for low values of *μ*_Y_, and too low coverage for large values. LS_exp_ provided too low coverage both for low and high values (Table 4).

**Table 4:** Actual coverage of the 95% confidence interval for *μ*_Y_ based on the sample-specific standard error (results from simulation with *n* = 108 observations and *r* = 10,000 replicates).

Expected value	Coverage ^1^					
*μ*_Y_	LS_lin_	WLS	ML_LN_	GLM_G_	GLM_N_	LS_exp_
1.714	0.98	0.94	0.95	0.93	0.94	0.83
2.164	0.99	0.95	0.95	0.93	0.94	0.95
2.614	0.96	0.95	0.95	0.93	0.94	0.93
2.568	0.97	0.95	0.95	0.93	0.94	0.90
3.018	0.94	0.95	0.95	0.93	0.93	0.83
3.468	0.92	0.95	0.95	0.93	0.94	0.94
3.422	0.93	0.95	0.95	0.92	0.93	0.93
3.872	0.89	0.94	0.95	0.93	0.94	0.95
4.322	0.89	0.94	0.94	0.93	0.94	0.87

^1^ Proportion of replicates where 95% confidence interval covers true expected value *μ*_Y_.

### 3.2. Application of the Regression Methods to the DIWA Dataset

The DIWA dataset consists of data from approximately 600 women for which a large amount of data, related to diabetes and obesity, were collected. Descriptive statistics for CRP, waist circumference and HOMA-IR are presented in Table 5, separate for each glucose tolerance group. 

**Table 5:** Descriptive statistics for C-reactive protein (CRP), insulin resistance (HOMA-IR) and waist circumference.

**Group**		**CRP**			**HOMA-IR**			**Waist circumference (cm)**		
	*n*	Mean	Median	SD	Mean	Median	SD	Mean	Median	SD
NGT ^1^	185	2.107	1.184	2.550	1.141	0.960	0.647	88.295	88.50	8.948
IGT ^1^	195	2.583	1.380	3.783	1.816	1.430	1.268	92.677	92.50	11.882
DM ^1^	218	4.468	1.856	10.255	4.677	2.835	5.842	98.083	98.00	12.631

^1^ Results for women with normal glucose tolerance (NGT), impaired glucose tolerance (IGT) and diabetes mellitus (DM).

#### 3.2.1. Regression Models for C-Reactive Protein (CRP) and Insulin Resistance (HOMA-IR)

**Figure 1:**
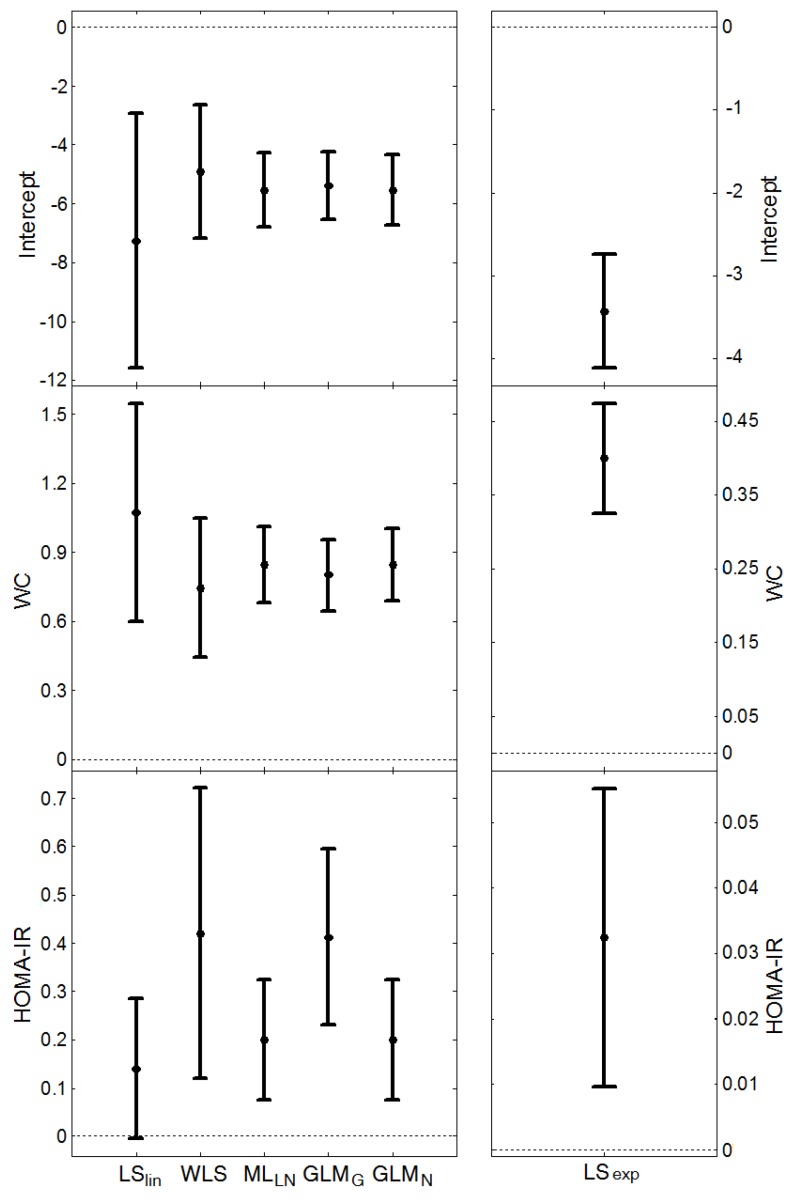
The parameter estimates and 95% confidence intervals for the different regression methods, when estimating CRP as a function of waist circumference (WC) and HOMA-IR, using *n* = 598 observations.

For CRP, the start model in the multivariable regression analysis included smoking, physical activity, waist circumference (WC), insulin resistance (HOMA-IR) and glucose tolerance (GT), where GT was classified into three categories: normal glucose tolerance, impaired glucose tolerance and diabetes mellitus. We used a model that allowed for different associations for the GT groups, by including the interaction terms WC∙DM and WC∙IGT. The final model, based on backward elimination using ML_LN_, contained WC and HOMA-IR, but no interaction term, thereby implying that the association with WC could be similar for the three GT groups (Figure 1).

**Figure 2:**
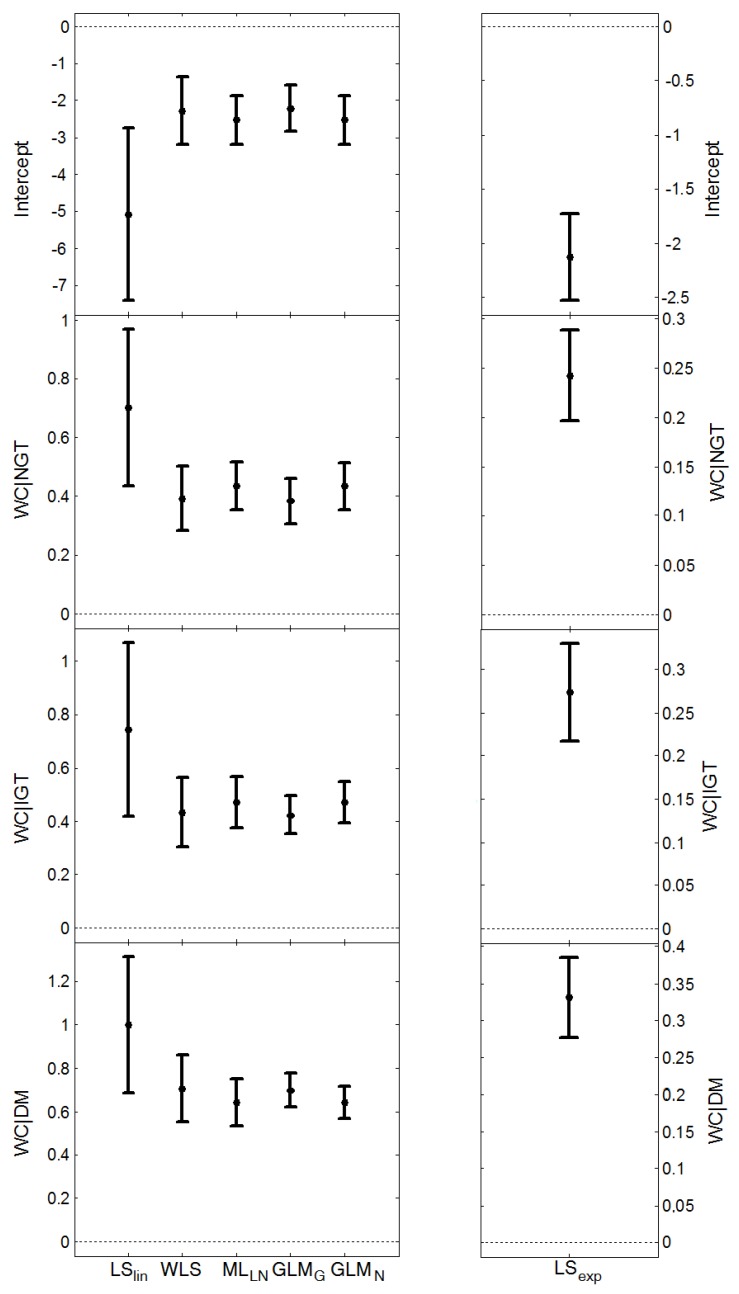
The parameter estimates and 95% confidence intervals for the different regression methods, when estimating HOMA-IR as a function of waist circumference (WC) and the interaction between WC and glucose tolerance group (normal glucose tolerance, impaired glucose tolerance and diabetes mellitus), using *n* = 598 observations.

For HOMA-IR, the start model in the multivariable regression analysis included WC, physical activity and smoking, and we allowed for possible different association with WC for the different glucose groups by including the interaction between waist circumference and glucose tolerance. The final model, based on backward elimination using ML_LN_, contained WC and the interaction between WC∙GT, thus allowing different WC parameters for each GT group (Figure 2). 

The estimated standard deviation, 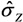, and the average length of the confidence intervals for *μ*_Y_, (estimated from the models presented in Figure 1 and Figure 2), are given in Table 6. ML_LN_, GLM_N_ and LS_exp_ gave similar estimates of *σ*_Z_ (this parameter cannot be estimated by LS_lin_). WLS provided the largest estimate whereas GLM_G_ gave the smallest. ML_LN_ and GLM_G_ had similar confidence intervals for the expected value, *μ*_Y_, GLM_N_ had the shortest intervals, whereas LS_lin_ had the longest intervals.

**Table 6:** The σ_Z_-estimates and mean length of 95% confidence intervals for *μ*_Y_, for CRP and HOMA-IR, *n* = 598.

**Method**	**CRP**			**HOMA-IR**	
	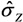	Length CI (mean, SD)		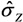	Length CI (mean, SD)
LS_lin_	-	1.61 (0.89)		-	1.10 (0.19)
WLS	1.22	1.51 (2.07)		0.73	0.64 (0.35)
ML_LN_	1.04	0.82 (0.86)		0.61	0.43 (0.19)
GLM_G_	0.71 (0.974 ^1^)	0.85 (1.26)		0.33 (2.52 ^1^)	0.47 (0.26)
GLM_N_	1.04	0.43 (0.23)		0.61	0.23 (0.06)
LS_exp_	1.04	1.19 (5.40)		0.60	0.50 (0.45)

^1^ Estimated scale parameter

#### 3.2.2. Quantification of Factors Associated with CRP and HOMA-OR (Method Comparison)

All of the methods demonstrated that WC was a significant predictor for CRP. According to the absolute-effects methods (LS_lin_, WLS, GLM_G_, GLM_N_ and ML_LN_), the CRP was expected to increase about 1 mg/L (between 0.74 and 1.07 mg/L) for every 10 cm in WC and, according to the relative-effects method (LS_exp_), the expected increase was 49% for every 10 cm in WC (exp(0.40) – 1 = 0.49), Figure 1. All methods showed a positive association between HOMA-IR and CRP. The expected increase in CRP was between 0.12 and 0.42 mg/L for every unit increase of HOMA-IR in the absolute-effects methods and 3% per unit of HOMA-IR for the relative-effects method. The association with HOMA-IR was not significant for LS_lin_ and very high for GLM_G_ and WLS (0.41 and 0.42, respectively). The point estimates from all methods had the same sign and for the absolute-effects methods the confidence intervals for β_WC_ overlapped, as did the intervals for β_HOMAIR_, Figure 1.

All methods found a positive association between HOMA-IR and WC in all glucose tolerance groups, Figure 2. Further, the results showed that women with DM had a significantly stronger association with WC than women with NGT, and this was significant for all methods. The results also indicated a stronger association with WC for women with IGT, compared to women with NGT; the interaction term for WC•IGT was significant for all absolute-effects methods except LS_lin_. Among the absolute-effects methods, HOMA-IR was expected to increase 0.64–1.00 per 10 cm WC for women with DM, 0.42–0.74 for women with IGT and 0.39–0.70 for women with NGT. The relative-effects method showed an expected increase in HOMA-IR of 39% per 10 cm for women with DM, 31% for women with IGT and 27% for women with NGT.

## 4. Discussion

Several methods for estimating a linear regression on log-normal data were compared. Much research has investigated making inferences, including confidence interval, of the expected value of a log-normal distribution, e.g. [36,37,38,39,40]. Here we considered the situation where the systematic part of the model for the outcome *Y* should be additive on the original scale (*μ*_YǀX_ = β_0_ + β_1_*X*_1_ + … + β*_p_X_p_*). Had we made the assumption that the systematic part was multiplicative, the regression coefficients could have been estimated either with a GLM using gamma distribution and the log link, or by a GLM using a normal distribution and identity link for *Z* = ln(*Y*), which give similar results [41,42]. But we wanted a model for estimating the absolute effect of each explanatory factor. In exposure assessment, we often want to assess the personal exposure to e.g., a specific compound in the air, by using a model that includes the important exposure determinants. Here the quantity is an important factor (e.g., time spent in different micro-environments, number of cigarettes smoked) and it is reasonable that the effect is linear. A linear model can also be used to estimate biologic interaction, discussed in Section 4.3 below.

Six methods were compared; four of them directly modeled the expected value of *Y* as a linear function of the explanatory variables, *μ*_YǀX_ = β_0_ + β_1_*X*_1_ + … + β*_p_X_p_* one method transformed the estimated coefficients, 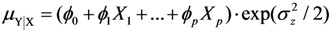 and finally the common method based on log-transformation was included for comparison, *μ*_ZǀX_ = δ_0_ + δ_1_*X*_1_ + … + δ*_p_X_p_*. Evaluation was made both using simulations and by applying the methods to a large data set to estimate well-known associations of abdominal adiposity (waist circumference, WC) on inflammation (measured using C-reactive protein, CRP) and insulin resistance (measured using HOMA-IR), respectively.

### 4.1. Method Comparison

In a simulation study we evaluated the regression methods in a situation where the expected outcome is a linear function of two explanatory variables. All methods except LS_exp_ provided unbiased estimates of the regression coefficients and the expected outcome, but the sample-specific standard error, 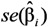, tended to be too small, thus overestimating the power. For LS_lin_, the assumption of a constant variance for *Y* resulted in confidence intervals for *μ*_Y_ with unnecessary high coverage for small *μ*_Y_-values and too low coverage at large *μ*_Y_-values. LS_exp_ does estimate the relative effect rather than the absolute and as a result the estimated expected values were biased and the coverage of the confidence intervals was erroneous. The confidence intervals from the GLM_G_ method had too low coverage, as a result of the underestimation of the variance 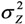. This is contrary to the situation with a multiplicative model, where the gamma distribution often provide reasonable estimates when applied to a log-normal variable [41,42]. ML_LN_, WLS and GLM_N_ provided approximately correct coverage, although GLM_N_ had a tendency to underestimate, as a result of using the estimate 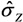, thus not including the stochastic variation of 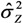 in the interval estimation. An approximate confidence interval taking into account its stochastic variation could be derived using Taylor expansion, see e.g. [43].

The methods were applied to two approximately log-normal response variables, CRP and HOMA-IR (almost 600 observations). The model for CRP contained WC and HOMA-IR, and the model for HOMA-IR contained WC and the interaction between WC and glucose tolerance groups (normal glucose tolerance [NGT], impaired glucose tolerance [IGT] and diabetes mellitus [DM]). When comparing confidence intervals for *β* and for *μ*_Y_, ML_LN_ and GLM_N_ consistently had narrower confidence intervals than WLS (and LS_lin_). From the simulation we saw that WLS tends to overestimate the variance. Because of underestimation of 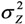, GLM_G_ had narrower intervals than ML_LN_ and GLM_N_ for *μ*_Y_, but from the simulation we know that the coverage will be too low. Thus ML_LN_ will have a higher power and for lognormal data the probability of detecting a true explanatory variable is higher. The smaller interval lengths of ML_LN_ corroborate the results of a previous simulation study [11]. 

### 4.2. Factors Associated with CRP and HOMA-IR, Respectively

Using all methods, the analysis demonstrated a significant positive association between CRP and WC. Associations between CRP and several measures of obesity and abdominal adiposity have been shown in a number of studies [44,45,46,47], and some studies indicate that abdominal adiposity has a stronger association with inflammation than total adiposity [48,49,50]. For CRP we could not find any significant interaction between glucose tolerance group and waist circumference, thus our results did not indicate that the association between obesity and the inflammation marker depends on the degree of glucose tolerance. Many studies have been based on only one or two of the GT groups, [24,51,52,53]. Our study showed an expected increase in CRP of between 0.74 and 1.07 mg/L per 10 cm increase in WC for the absolute-effects methods and 49% per 10 cm for the relative-effects method. All methods, with the exception of LS_lin_, showed a significant positive association between CRP and HOMA-IR. The lack of significant association using LS_lin_ can probably be explained by the estimates of the variance. In the LS_lin_ method the heteroscedasticity is not taken into account. 

In the analysis of HOMA-IR, all methods identified WC as a significant predictor for HOMA-IR. There was also a significant interaction between glucose tolerance group and waist circumference, thus the absolute-effects models showed a departure from additivity. These results cannot be interpreted causally, but the interaction indicates that obesity might affect insulin resistance more for women who have diabetes mellitus compared to those with normal glucose tolerance. All models methods found a significantly stronger WC-association for women with DM compared to women with NGT, and all methods (apart from LS_lin_) also had a significantly stronger WC-association for women with IGT compared to NGT. From the simulation we know that LS_lin_ has larger standard errors than the other methods and thus lower power. The relative-effects method LS_exp_ also showed a significant interaction between glucose tolerance group and waist circumference, *i.e.*, departure from multiplicativity.

Even if HOMA-IR typically has a skewed non-normal distribution, regression analyses have been performed using both untransformed and log-transformed HOMA-IR values, see [54,55] shows an expected increase in HOMA-IR with 3.5 units per 10 cm WC, using LS_lin_ on persons with DM, to be compared with 0.64–1.00 units in our study. The difference in association might be explained by the fact that the previous study included both men and women of different ages [56] uses the method here denoted LS_exp_ and finds a positive association; about 22% per 10 cm WC, while we found the association to be stronger; 27%–39%.

### 4.3. Model Choice

The choice between an additive or multiplicative model affects the interpretation of the estimated coefficients. The aim of a regression analysis might be simply to test whether there is a significant association between an outcome and a potential explanatory variable. Another aim can be to quantify a specific association (e.g., the absolute or relative effect), or assess the biologic interaction. If the study is purely exploratory, using epidemiological data, residual analysis can be used to decide which model that fits the data best. The model choice might be based on previous knowledge, e.g., about the biological process, from experimental studies.

In risk-modeling, a log-linear model is often used, *φ*(*Z*, *β*) = exp(*α*_0_ + *α_1_X_1_* + … + *α_k_X_k_ + βZ*), where φ can be the odds ratio or rate ratio function, *X_1_-X_k_* are covariates and *Z* is the exposure variable of interest. In this model the ratio has an exponential dependence on *Z*; exp(β*Z*). However, linear models have also been discussed, see [57], for example in radiation epidemiology, where the linear relative rate model *φ*(*Z*,*β*) = exp(*α*_0_ + *α_1_X_1_* + … + *α_k_X_k_*)(1–*βZ*) allows the rate ratio to increase linearly with the dose *Z* [58]. 

Not only the main effects but also potential interactions can be of interest. Interaction in a statistical sense is scale dependent, e.g., an absence of interaction in absolute-scale will lead to interaction in log-scale. An interaction in a linear absolute-effects model is additive, while an interaction in a log-linear relative-effects model is multiplicative. In epidemiology, an additive interaction (effect-modification on the absolute scale) is often considered more important when assessing public health impact, and seems to correspond more to biologically based notions of interaction [9,59,60]. There is a need for regression methods that can assess biologic interaction, as discussed in several articles. In logistic regression it is implicit that we have a multiplicative statistical relation and if an additive biological model holds, the logistic analysis would require three parameters to summaries the joint effects of only two variables, [61]. Additive interactions are given directly in a linear model, however a logistic regression model can be defined in such a way that additive interactions (e.g., biologic interaction) can be assessed [62].

### 4.4. Strengths and Weaknesses

Five regression methods for estimating associations on the absolute scale of the explanatory variables were compared, with regard to bias and standard deviation for the estimated coefficients and also with regard to the estimated expected outcome and its confidence interval. In addition, the standard method for log-normal data (log-transformation) was evaluated. The comparison of the methods was made both in a simulation study and using two examples. The absolute-effects methods provide similar results for the association with the predictors for CRP and HOMA-IR, respectively. The results from the examples are consistent with those from the simulations. 

The aim of this study was not to provide a complete statistical model of which factors that are associated with CRP and HOMA-IR, but to compare the statistical methods. The number of factors in the regression models was therefore kept small; the simulation model only included two explanatory variables and in the models for CRP and HOMA-IR, only those variables that were significant after backward elimination using ML_LN_ were included. Thus, all factors were significant for ML_LN_ (and also for GLM_N_). This could be seen as an advantage for these methods, compared to for example a situation in which LS_exp_ had been used to select the model. However, since we assume a linear model (*i.e.*, absolute effects) it is natural to use a method that can estimate the absolute effects in the model selection process. We also wanted the method that was expected to have a high power, and based on previous studies, [11], ML_LN_ was expected to have higher power than e.g., WLS and LS_lin_.

## 5. Conclusions

In medical research we often want to identify and quantify associations using regression analysis. Log-normal data are common and there are situations when the absolute effects are of interest (rather than the relative) and thus there is a need for linear regression methods on untransformed log-normal data. We have evaluated several regression methods using both large scale simulations of personal exposure to PM, and by applying the methods to data on biomarkers (CRP and HOMA-IR). The LS_exp_ does not provide estimates of the absolute effects and the expected outcome can be biased. The LS_lin_ and GLM_G_ provide correct point estimates of the expected outcome, but confidence intervals with incorrect coverage. The ML_LN_ and GLM_N_ worked best (unbiased estimates, narrow confidence intervals), although ML_LN_ tends to have a slightly more correct coverage for the confidence intervals.
